# Correlates of Peripheral Blood Mitochondrial DNA Content in a General Population

**DOI:** 10.1093/aje/kwv175

**Published:** 2015-12-24

**Authors:** Judita Knez, Ellen Winckelmans, Michelle Plusquin, Lutgarde Thijs, Nicholas Cauwenberghs, Yumei Gu, Jan A. Staessen, Tim S. Nawrot, Tatiana Kuznetsova

**Keywords:** general population, mitochondrial DNA, peripheral blood

## Abstract

Accumulation of mitochondrial DNA (mtDNA) mutations leads to alterations of mitochondrial biogenesis and function that might produce a decrease in mtDNA content within cells. This implies that mtDNA content might be a potential biomarker associated with oxidative stress and inflammation. However, data on correlates of mtDNA content in a general population are sparse. Our goal in the present study was to describe in a randomly recruited population sample the distribution and determinants of peripheral blood mtDNA content. From 2009 to 2013, we examined 689 persons (50.4% women; mean age = 54.4 years) randomly selected from a Flemish population (Flemish Study on Environment, Genes, and Health Outcomes). Relative mtDNA copy number as compared with nuclear DNA was measured by quantitative real-time polymerase chain reaction in peripheral blood. There was a curvilinear relationship between relative mtDNA copy number and age. mtDNA content slightly increased until the fifth decade of life and declined in older subjects (*P*_age_^2^ = 0.0002). mtDNA content was significantly higher in women (*P* = 0.007) and increased with platelet count (*P* < 0.0001), whereas it was inversely associated with white blood cell count (*P* < 0.0001). We also observed lower mtDNA content in women using estroprogestogens (*P* = 0.044). This study demonstrated in a general population that peripheral blood mtDNA content is significantly associated with sex and age. Blood mtDNA content is also influenced by platelet and white blood cell counts and estroprogestogen intake. Further studies are required to clarify the impact of chronic inflammation and hormone therapy on mitochondrial function.

Mitochondrial dysfunction is implicated in aging (1, 2) and in pathological processes, such as carcinogenesis (3) and inflammation (4, 5). Mammalian cells contain different numbers of mitochondria, ranging from hundreds to several thousand (6). Within cells, mitochondria are not autonomous but instead are organized into a dynamic network, interacting with each other through processes of fusion and fission (7). Their number and shape constantly change in response to energy demands, oxidative stress, and pathological conditions (8). A single mitochondrion contains on average 2–8 copies of circular mitochondrial DNA (mtDNA) molecules (9). The mitochondrial-to-nuclear genome ratio (mtDNA content) in tissues and body fluids correlates with the size and number of mitochondria (10). Limited repair capacity and a lack of histons and noncoding introns, along with the immediate proximity of reactive oxygen species, make mtDNA susceptible to mutations. Accumulation of mtDNA mutations is counteracted by intermitochondrial interaction and mitophagy (7, 11). Nonetheless, when present in excess, mtDNA mutations might lead to alterations of mitochondrial biogenesis and function that can result in a decrease of mtDNA content within cells (12).

Experimental studies in mice have shown that defective mtDNA replication accelerates aging and reduces life span (2). Furthermore, a similar decrease in mtDNA content in different kinds of cells, including myocardial and hematopoietic cells, was demonstrated in rapidly aging mice (1). On the other hand, in humans, the mtDNA content in peripheral blood has been found to be associated with overall level of oxidative stress (13) and increased risks of colorectal cancer (3), breast cancer (14), type 2 diabetes (15), and cardiovascular disease (16). This implies a possible role of mtDNA content as a potential biomarker in processes associated with oxidative stress and inflammation. However, data on correlates of mtDNA content in a general population are sparse. Therefore, our objectives in the present study were to describe, in a randomly recruited population sample, the distribution and determinants of peripheral blood mtDNA content.

## METHODS

### Study participants

The Flemish Study on Environment, Genes, and Health Outcomes (FLEMENGHO) is a large, ongoing family-based Belgian population study. The Ethics Committee of the University of Leuven (Leuven, Belgium) approved the FLEMENGO study protocol. Participants gave informed consent. From August 1985 to December 2002, we identified a random population sample, stratified by sex and age, from a geographically defined area in northern Belgium. Households, defined as people who lived at the same address, were the sampling unit. We numbered households consecutively and generated a random number list by means of the SAS random function (SAS Institute, Inc., Cary, North Carolina). Households with a number matching the list were invited to participate; household members aged ≥18 years were eligible.

From 3,324 invited subjects, 2,593 initially agreed to participate in the study (response rate 78%). From 2009 to 2013, we invited 919 former participants to a regular follow-up examination at our local examination center. Of those, 737 renewed their consent (response rate 80%). We excluded 48 participants from analysis, because blood for DNA extraction was not available (*n* = 5) or because the DNA (*n* = 32) or the quantitative polymerase chain reaction (PCR) procedure (*n* =11) for measurement of mtDNA content was of insufficient quality. Thus, the number of participants statistically analyzed totaled 689.

### Clinical measurements

On the day of the examination, participants completed a validated questionnaire inquiring into lifestyle, medical history, and intake of medications. When possible, the diseases reported via the questionnaires were verified against the medical records of general practitioners or hospitals. The diseases were coded using the *International Classification of Diseases, Eighth Revision*. In the Web Appendix (available at http://aje.oxfordjournals.org/), we provide detailed information on disease codes and medications which we included in the sensitivity analyses. The questionnaire also obtained detailed information on menstrual status and use of hormones for contraception or postmenopausal systemic hormone therapy (SHT). Trained nurses measured anthropometric characteristics and blood pressure 5 times consecutively to the nearest 2 mm Hg after the participant had rested for 5 minutes in the sitting position. Hypertension was defined as a blood pressure of at least 140 mm Hg systolic or 90 mm Hg diastolic or use of antihypertensive medication. Body mass index was defined as weight in kilograms divided by squared height in meters. A differential blood cell count was performed using an automated analyzer.

### Measurement of mtDNA content

Genomic DNA was extracted from peripheral blood using the QIAmp DNA Mini Kit (QIAGEN GmbH, Hilden, Germany), following the manufacturer's instructions. The concentration and purity of the extracted DNA were determined using a Nanodrop spectrophotometer (ND-1000; Isogen Life Science B.V., De Meern, the Netherlands). The DNA samples were diluted to 2.4 ng/µL. To measure mtDNA content, we used real-time quantitative PCR, as described previously (17). We determined the relative ratio of 2 mtDNA sequences (mitochondrially encoded NADH dehydrogenase 1 (*MT-ND1*) and mitochondrial forward primer from nucleotide 3212 and reverse primer from nucleotide 3319 (*MTF3212/R3319*)) to a single housekeeping nuclear gene (acidic ribosomal phosphoprotein P0 (*RPLP0*)). The master mix contained Fast SYBR Green dye 2x (Applied Biosystems, Inc., Foster City, California), forward and reverse primers diluted to 300 nM per well, and RNAse-free water. Primer sequences for the selected amplification targets, with their corresponding efficiencies, are listed in Web Table 1. Analyses were run in triplicate on MicroAmp Optical 384-well reaction plates (Applied Biosystems, Inc.). A single well contained 2.5 µL of the diluted DNA sample and 7.5 µL of the master mix. In addition, each plate had 6 interrun calibrators and 2 no-template controls to test for contamination. We amplified the target sequences in a 7900HT Fast Real-Time PCR thermal cycler (Applied Biosystems, Inc.). The thermal cycling profile was 20 seconds at 95°C for activation of the polymerase, followed by 40 cycles of 1 second at 95°C for denaturation and 20 seconds at 60°C for annealing and extension. A melting curve analysis was performed after each run to confirm the absence of nonspecific products. Cycle threshold (*C_t_*) values of the 2 mitochondrial DNA sequences were normalized relative to the nuclear gene using qBase quantification software (Biogazelle NV, Zwijnaarde, Belgium). A detailed description of the calculation of mtDNA content is provided in the Web Appendix. Briefly, the qBase software uses the relative normalized values based on the ΔΔ*C_t_* method, taking multiple sequences and the interrun calibrators into account (18). The coefficients of variation for triplicate measurements within the same run were 0.44%, 0.41%, and 0.30% for *MT-ND1*, *MTF3212/R3319*, and *RPLP0*, respectively. The coefficient of variation for the interrun samples was 4.66%.

### Statistical analysis

For database management and statistical analysis, we used SAS software, version 9.3. We tested the normality of the mtDNA distribution by computing skewness and kurtosis coefficients and by applying the Kolmogorov-Smirnov test. The central tendency and spread of the data are reported as mean values with standard deviations (SDs). We compared means and proportions using the *t* test and the χ^2^ test, respectively. Significance was defined as *P* < 0.05 in a 2-sided test. We performed stepwise multiple regression to assess the independent correlations of mtDNA content with sex, age, body height, body weight, body mass index, waist circumference, systolic and diastolic blood pressures, white blood cell (WBC) count, platelet count, plasma glucose, serum insulin, total cholesterol, serum creatinine, current smoking, alcohol consumption, treatment with antihypertensive or lipid-lowering drugs, and SHT. We set the *P* values for individual variables to enter and to stay in the regression models at 0.10. We conducted regression diagnostic analyses (variance inflation factors) to exclude possible collinearity. We also tested the association of important covariables with mtDNA content by use of a mixed model. This technique allows accounting for covariables as well as for the nonindependence of observations within families. We expressed multivariable-adjusted effect sizes for a 1-SD increase in the explanatory variable. We expressed the magnitude of mtDNA changes in absolute values and as a fraction of the SD of mtDNA content.

## RESULTS

### Characteristics of participants

The 689 Caucasian participants included 347 (50.4%) women, 338 (49.1%) hypertensive patients, and 16 (2.3%) diabetic patients. The mean age was 54.4 (SD, 15.2) years, and ages ranged from 18 years to 89 years. Table 1 summarizes the clinical and biochemical characteristics of the participants by sex. Compared with women, men had higher diastolic blood pressure, blood glucose levels, and serum creatinine levels. Histories of blood disease, cancer, alcohol consumption, and antiaggregation therapy were also more frequently reported in men. On the other hand, women had higher total serum cholesterol levels. Platelet and lymphocyte counts were higher in women, whereas monocyte count was higher in men. Among women, 221 (63.7%) were in menopause and 57 (16.4%) reported SHT use (Table 1).

**Table 1.** Characteristics of Participants, by Sex, in the Flemish Study on Environment, Genes, and Health Outcomes, 2009–2013

Characteristic	Clinical Measurement						*P* Value
	Women (*n* = 347)			Men (*n* = 342)			
	Mean (SD)	No.	%	Mean (SD)	No.	%	
Anthropometric factors							
Age, years	54.8 (14.8)			54.0 (15.7)			0.47
Body mass index^a^	26.9 (4.8)			27.6 (4.1)			0.052
Systolic blood pressure, mm Hg	131.0 (18.6)			132.6 (14.9)			0.22
Diastolic blood pressure, mm Hg	80.8 (9.5)			83.6 (9.8)			0.0002
Questionnaire data							
Current smoking		58	16.7		53	15.5	0.66
Current alcohol drinking		74	21.3		181	52.9	<0.0001
Hypertensive		156	45.0		182	53.2	0.030
Treated for hypertension		103	29.7		112	32.8	0.39
Diabetes		5	1.44		11	3.22	0.12
Antiaggregation therapy^b^		44	12.7		65	19.0	0.023
History of inflammatory disease^b^		14	4.03		16	4.68	0.68
History of cancer^b^		10	2.88		17	4.97	0.16
History of blood disease^b^		6	1.73		36	10.5	<0.0001
Menopause		221	63.7				
Systemic hormone therapy		57	16.4				
Substitution		18	5.19				
Contraception		39	11.2				
Estrogen		11	3.17				
Combined		46	13.3				
Biochemical data							
Plasma glucose, mmol/L	4.89 (0.78)			5.03 (0.81)			0.016
Serum creatinine, µmol/L	79.8 (14.9)			97.8 (25.1)			<0.0001
Serum total cholesterol, mmol/L	5.23 (0.98)			4.84 (0.88)			<0.0001
Blood cell count, ×10^9^ cells/L							
Platelets	252.4 (59.1)			213.9 (47.0)			<0.0001
White blood cells	6.52 (1.72)			6.31 (1.47)			0.086
Lymphocytes	2.10 (0.68)			1.87 (0.61)			<0.0001
Segmented neutrophils	3.73 (1.29)			3.70 (1.03)			0.76
Monocytes	0.51 (0.15)			0.55 (0.18)			0.0006
Eosinophils	0.15 (0.11)			0.16 (0.11)			0.44
Basophils	0.03 (0.02)			0.03 (0.02)			0.21
mtDNA content^c^	1.07 (0.37)			0.98 (0.32)			0.0008

Abbreviations: mtDNA, mitochondrial DNA; SD, standard deviation.

^a^ Weight (kg)/height (m)^2^.

^b^ A detailed description of medications and disease codes is provided in the Web Appendix.

^c^ Relative ratio of copy numbers of 2 mtDNA sequences (mitochondrially encoded NADH dehydrogenase 1 (*MT-ND1*) and mitochondrial forward primer from nucleotide 3212 and reverse primer from nucleotide 3319 (*MTF3212/R3319*)) to a single housekeeping nuclear gene (acidic ribosomal phosphoprotein P0 (*RPLP0*)).

### mtDNA content and its determinants

**Figure 1.**
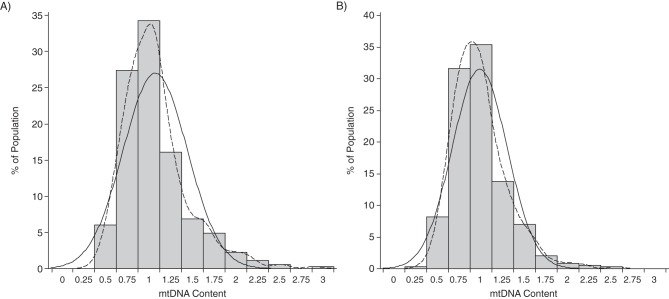
Distribution of the relative mitochondrial DNA (mtDNA) content (ratio of copy numbers) among women (A) and men (B) in the Flemish Study on Environment, Genes, and Health Outcomes, 2009–2013. The curves represent the fitted normal (full line) and Kernel (dashed line) density plots. In women, the coefficients of skewness and kurtosis were 1.37 (*P* < 0.01) and 2.94, respectively. In men, the coefficients of skewness and kurtosis were 1.18 (*P* < 0.01) and 2.61, respectively.

Relative mtDNA content (ratio of copy numbers (*MT-ND1* +*MTF3212/R3319* to *RPLP0*) as described above) averaged 1.03 (95% confidence interval: 0.34, 1.72). Figure 1 and Web Figure 1 show the distribution of mtDNA content by sex and in the entire population, respectively. Women had a significantly higher mtDNA content than men (1.07 vs. 0.98; *P* = 0.0008) (Table 1). The distribution of mtDNA was positively skewed (*P* < 0.01), with a coefficient of skewness of 1.33.

**Figure 2.**
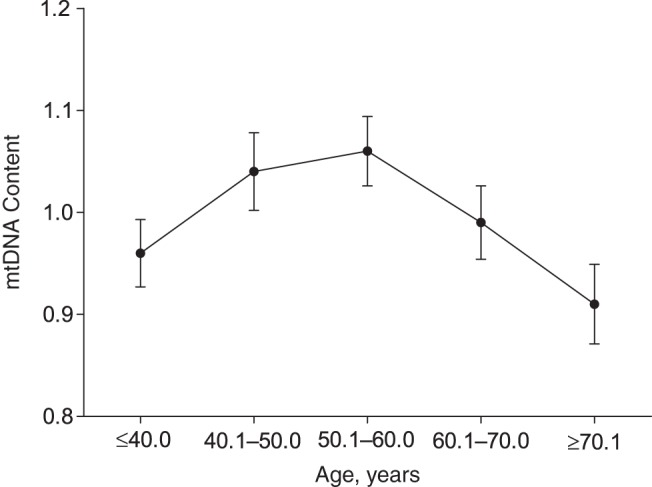
Mean mitochondrial DNA (mtDNA) content (ratio of copy numbers; see Methods), by age, in the Flemish Study on Environment, Genes, and Health Outcomes, 2009–2013. Results were adjusted for sex, white blood cell and platelet counts, use of systemic hormone therapy, and family clusters. The numbers of individuals in the age groups were: ≤40 years, *n* = 133; 40.1–50.0 years, *n* = 113; 50.1–60.0 years, *n* = 180; 60.1–70.0 years, *n* = 150; and ≥70.1 years, *n* =113. Bars, standard errors.

We noticed a curvilinear relationship between relative mtDNA content and age (Web Figure 2). Indeed, mtDNA content increased until the fifth decade of life and declined in older subjects (Figure 2). In the stepwise analysis, mtDNA content was significantly and independently associated with age only when we added age squared to the model (Table 2).

**Table 2.** Correlates^a^ of Mitochondrial DNA Content in the Flemish Study on Environment, Genes, and Health Outcomes, 2009–2013

Parameter	mtDNA Content^b^				
	Partial *r*^2^, %	Parameter Estimate	SE	95% CI	*P* Value
Age, per year^c^	2.28	0.018	0.005	0.008, 0.028	0.0005
Age^2^, years		−0.0002	0.00005	−0.0003, −0.0001	0.0002
Female sex	0.64	0.076	0.028	0.02, 0.13	0.007
White blood cell count (1.60 × 10^9^ cells/L)^d^	4.11	−0.093	0.013	−0.12, −0.068	<0.0001
Platelet count (56.8 × 10^9^ cells/L)^d^	3.41	0.056	0.014	0.029, 0.083	<0.0001
Use of systemic hormone therapy	0.46	−0.094	0.050	−0.19, 0.004	0.061
Total adjusted *R*^2^, %	10.9				

Abbreviations: CI, confidence interval; mtDNA, mitochondrial DNA; SE, standard error.

^a^ The covariables considered for entry into the stepwise regression model were sex, age, age^2^, body height, body weight, waist circumference, body mass index, systolic and diastolic blood pressure, plasma glucose, serum insulin, total cholesterol, serum creatinine, current smoking and alcohol drinking, and systemic hormone therapy. We set the *P* values for covariates to enter and to stay in the regression models at 0.10. Variance inflation factors were ≤1.30 for all explanatory variables.

^b^ Relative ratio of copy numbers of 2 mtDNA sequences (mitochondrially encoded NADH dehydrogenase 1 (*MT-ND1*) and mitochondrial forward primer from nucleotide 3212 and reverse primer from nucleotide 3319 (*MTF3212/R3319*)) to a single housekeeping nuclear gene (acidic ribosomal phosphoprotein P0 (*RPLP0*)).

^c^ Partial *r*^2^ for age includes the partial *r*^2^ for age^2^.

^d^ Parameter estimates and corresponding SEs and 95% CIs for blood cell counts are expressed for a 1-standard-deviation increase in the explanatory variable.

When adjusting for age and age squared, mtDNA content was higher in women (*P* = 0.007) and was positively associated with platelet count (*P* < 0.0001), whereas it decreased with WBC count (*P* < 0.0001; Table 2). Overall, the WBC counts (as compared with platelet counts) explained slightly more variance in mtDNA (difference in partial *r*^2^ = 0.7%). Use of SHT was borderline-associated with a decrease in mtDNA content (*P* = 0.06). The explained variance totaled 10.9% for the mtDNA content.

In the sensitivity analyses, our findings remained consistent after exclusion of participants on antiaggregation therapy (*n* = 109; Web Table 2) or participants with a history of blood diseases (*n* = 47; Web Table 3), inflammatory diseases (*n* = 30; Web Table 4), or cancer (*n* = 27; Web Table 5). We also performed additional sensitivity analysis selecting only healthy participants (*n* = 306) without histories of diabetes, hypertension, coronary heart disease, inflammatory disorders, blood diseases, or cancer. The findings on correlates remained constant, although they were somewhat attenuated because of the smaller number of subjects included in this analysis (Web Table 6). Moreover, the associations of mtDNA content with sex and SHT did not reach significant levels, but trends were similar.

### Association between mtDNA content and blood cell counts

**Figure 3.**
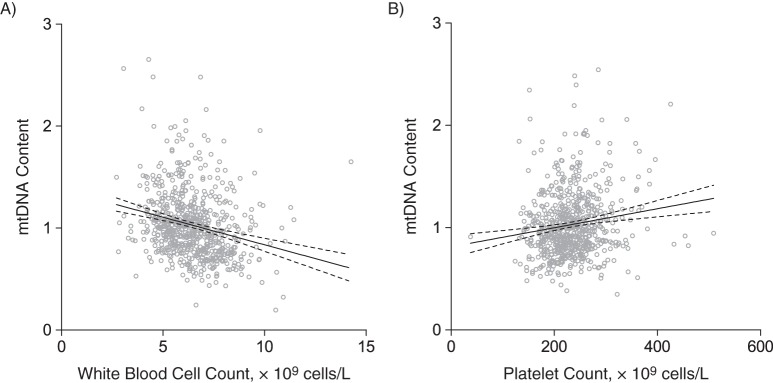
Relative mitochondrial DNA (mtDNA) content (ratio of copy numbers; see Methods) according to white blood cell (WBC) count (A) (*r* = −0.24, *P* = <0.0001) and platelet count (B) (*r* = 0.15, *P* = <0.0001) in multivariable-adjusted analyses in the Flemish Study on Environment, Genes, and Health Outcomes, 2009–2013. The solid and dashed lines represent the regression line and the 95% confidence interval, respectively. Analyses were adjusted for age, age^2^, sex, use of systemic hormone therapy, and family clusters. In addition, for WBC count, the adjusted model included platelet count. For platelet count, the adjusted model included WBC count.

We further investigated the association between mtDNA content and blood cell counts in multivariable-adjusted analyses, while accounting for family clusters and important covariables selected by means of the stepwise model. For a 1-SD increment in platelet (56.8 × 10^9^ cells/L) and WBC (1.60 × 10^9^ cells/L) counts, the mtDNA content increased by 0.057 (or by 0.16 SD of mtDNA content; *P* < 0.0001) and decreased by 0.096 (0.27 SD of mtDNA content; *P* < 0.0001), respectively (Table 3). Figure 3 further illustrates the correlation between the adjusted mtDNA content and WBC and platelet counts. Partial Pearson's correlation coefficients were −0.24 and 0.15, respectively (*P* < 0.0001; Figure 3). Moreover, we found decreases in mtDNA content of 0.090 (0.26 SD), 0.051 (0.15 SD), and 0.051 (0.15 SD) for a 1-SD increase in the number of segmented neutrophils, monocytes, and lymphocytes, respectively (*P* ≤ 0.0002; Table 3). Web Figure 3 illustrates the correlations between the adjusted mtDNA content and different types of WBCs. In our sensitivity analyses, the results were consistent after exclusion of participants with a history of blood diseases (Web Table 7).

**Table 3.** Multivariable-Adjusted^a^ Correlations of Mitochondrial DNA Content With Blood Cell Counts in the Flemish Study on Environment, Genes, and Health Outcomes, 2009–2013

Blood Cell Count	mtDNA Content^b^			
	Parameter Estimate	SE	95% CI	*P* Value
Platelets (56.8 × 10^9^ cells/L)	0.057	0.014	0.031, 0.085	<0.0001
White blood cells (1.60 × 10^9^ cells/L)	−0.096	0.013	−0.12, −0.069	<0.0001
Segmented neutrophils (1.17 × 10^9^ cells/L)	−0.090	0.013	−0.12, −0.064	<0.0001
Monocytes (0.17 × 10^9^ cells/L)	−0.051	0.014	−0.078, −0.024	0.0002
Lymphocytes (0.66 × 10^9^ cells/L)	−0.051	0.014	−0.079, −0.024	0.0002
Eosinophils (0.11 × 10^9^ cells/L)	−0.0012	0.013	−0.026, 0.024	0.92
Basophils (0.017 × 10^9^ cells/L)	−0.015	0.013	−0.041, 0.011	0.25

Abbreviations: CI, confidence interval; mtDNA, mitochondrial DNA; SE, standard error.

^a^ Results were adjusted for sex, age, age^2^, systemic hormone therapy, and family clusters. For white blood cells, the adjusted model included platelet count. For platelets, the adjusted model included white blood cell count.

^b^ Relative ratio of copy numbers of 2 mtDNA sequences (mitochondrially encoded NADH dehydrogenase 1 (*MT-ND1*) and mitochondrial forward primer from nucleotide 3212 and reverse primer from nucleotide 3319 (*MTF3212/R3319*)) to a single housekeeping nuclear gene (acidic ribosomal phosphoprotein P0 (*RPLP0*)). Parameter estimates and corresponding SEs and 95% CIs are expressed for a 1-standard-deviation increase in the explanatory variable.

### Association between mtDNA content, sex, and SHT

**Figure 4.**
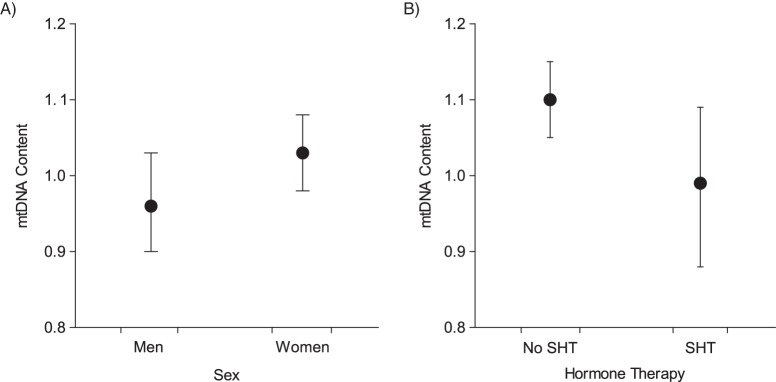
Multivariable-adjusted relative mitochondrial DNA (mtDNA) content (ratio of copy numbers; see Methods), by sex (A) and use of systemic hormone therapy (SHT) (B), among women in the Flemish Study on Environment, Genes, and Health Outcomes, 2009–2013. Results were adjusted for sex, age, age^2^, white blood cell and platelet counts, and family clusters. In part A, the *P* value for the difference between men (*n* = 342) and women (*n* = 347) was 0.013. In part B, the *P* value for the difference between women using SHT (*n* = 290) and women not using SHT (*n* = 57) was 0.044.

In multivariable-adjusted analyses, mtDNA content remained significantly higher in women than in men (1.03 vs. 0.96; *P* = 0.013) (Figure 4A). Notably, women on SHT had lower adjusted mtDNA content than women who did not report use of SHT (0.99 vs. 1.10; *P* = 0.044) (Figure 4B). As illustrated in Web Figure 4, we observed the same trend of a lower mtDNA content in women who used hormones for contraception or postmenopausal substitution therapy.

## DISCUSSION

The present study investigated the distribution and determinants of peripheral blood mtDNA content in a randomly selected general population sample. First, we demonstrated the curvilinear association of mtDNA content with age. Second, in our study, we found an increase in mtDNA content with higher platelet count and a decline in mtDNA content with higher WBC count. Third, in multivariable-adjusted analysis, we observed a higher level of mtDNA content in women compared with men. Moreover, we found a slight decrease in mtDNA content in women using SHT.

Previously published experimental studies showed differences in mitochondrial function between female and male rats (19, 20). Notably, female rats were less prone to mtDNA injury by reactive oxygen species (19). Recently, López et al. (21) confirmed the presence of sex-specific regulation of mtDNA levels in humans as well. In 386 subjects, a sex-specific linkage analysis showed that separate quantitative trait loci are involved in the control of peripheral blood mtDNA content in women and men. Moreover, in line with our observations, a recent study in 1,088 subjects of European descent showed higher peripheral blood mtDNA content in women compared with men (22). The same sex difference was also reported in patients with renal cell carcinoma (23) and colorectal cancer (24) and in healthy subjects (25, 26). On the other hand, some published reports have failed to demonstrate differences in mtDNA content between men and women (3, 21, 27).

An experimental study in mice demonstrated that defective mtDNA replication mechanisms caused premature onset of aging-related phenotypes (2). Along similar lines, Sahin et al. (1) showed that aging was accompanied by a decrease in the mtDNA content of myocardial, hepatic, and hematopoietic cells. Recently, the correlation between telomere length and peripheral blood mtDNA content was also explored in humans. Most of the studies (28–30), albeit not all (31), found a higher mtDNA content in subjects with longer telomeres. Taken together, these observations imply a change in mtDNA content with aging. Nonetheless, studies of human blood mtDNA content have shown contradictory results. In line with our findings, 3 large epidemiologic studies and a case-control study found a significant decline in blood mtDNA content with older age (22, 25, 27, 32). Notably, higher mtDNA content in the elderly subjects was associated with better cognitive function, physical performance (27), and longevity (32). Similar to our observations, Mengel-From et al. (27) also described a nonlinear relation between mtDNA content and age. On the other hand, several studies in cancer patients did not take this nonlinear association into account and therefore failed to demonstrate a significant association between mtDNA and age (3, 14, 23, 24).

In addition to sex and age, we demonstrated that blood cell counts are other important covariables influencing mtDNA content. The relative mtDNA content in peripheral blood depends largely on platelet and WBC counts (33). Mitochondrial function in both types of blood cells changes in response to pathological conditions (34, 35). For instance, systemic stress due to hyperglycemia induces overproduction of mitochondrial reactive oxygen species in human platelets (35, 36). On the other hand, because platelets have no nucleus and therefore reference total DNA is not increasing with higher platelet count, it is likely that the relative amount of mtDNA increases with the amount of platelets. Future studies might consider platelet count as an important covariable of mtDNA content measured in all cells derived from peripheral blood.

WBC count is an established marker of inflammation. In a study by Bartz et al. (37), metabolic changes occurring during inflammation in mice caused a decrease in mtDNA content and activated mechanisms to restore it to normal levels. Moreover, in a study by Zhou et al. (5), increased production of reactive oxygen species by mitochondria triggered the assembly of multiprotein inflammatory complexes called inflammasomes. Taken together, inflammation can damage mtDNA, and this might further stimulate the inflammation process. In previous clinical studies, both peripheral blood WBC count and mtDNA content were associated with inflammation-related processes, such as atherosclerosis and diabetes (15, 16, 38, 39). To our knowledge, only 1 small cross-sectional study of 40 benzene-exposed workers and 40 controls found a negative relationship between peripheral blood mtDNA level and WBC count (*r* = −0.22; *P* = 0.05) (40). WBCs are a versatile group of cells, and the precise molecular mechanisms governing their mitochondrial network dynamics remain to be established. Therefore, the link between WBC count, mtDNA levels, and oxidative stress needs to be confirmed in future studies.

Replication of mtDNA and mitochondrial biogenesis depend on mitochondrial- and nuclear-encoded proteins. The signaling pathways between the 2 cellular compartments are complex and not yet completely understood. Scarpulla (41) demonstrated that the nuclear transcription factor nuclear respiratory factor 1 (NRF-1) acts directly on genes that regulate mtDNA transcription. In human cells, treatment with estradiol increased the level of nuclear respiratory factor 1 and therefore induced mitochondrial biogenesis (42). However, estrogenic stimulation of cellular and mitochondrial proliferation results not only in the production of new DNA molecules but also in an increased risk of mutations during DNA replication (43). Moreover, several estrogen metabolites can cause alkylation and oxidative damage to cellular proteins and DNA (43). In line with these molecular mechanisms, large clinical trials and population studies have shown that therapy with sex hormones in women does not have only beneficial effects, but on the contrary can increase the risk of cardiovascular disease (44, 45) and cancer (46). In line with our results, López et al. (21) reported an association between mtDNA levels and use of oral contraceptives. However, a decline in mtDNA content with SHT was not described previously. Taking together, these findings might highlight the necessity for further studies on the impact of artificial sex hormones on human cells.

The present study must be interpreted within the context of its limitations and strengths. First, we measured mtDNA content in easily accessible peripheral blood buffy coat. The composition of the buffy coat might vary with regard to counts of platelets and WBC (33, 47). Nonetheless, all blood samples were processed following the same protocol, and mtDNA content was standardized to the amount of nuclear DNA to minimize sample-to-sample variation. Second, we assessed the general level of systemic inflammation using only WBC count.

In conclusion, we demonstrated in a general population sample that peripheral blood mtDNA content was significantly associated with sex and age. In addition, peripheral blood mtDNA content was influenced by platelet count, WBC count, and systemic intake of estroprogestogens. Future studies on mtDNA content in peripheral blood should take these covariates into account. Moreover, further molecular and population studies are required to clarify the impact of inflammation and hormone therapy on mitochondrial function.

## Supplementary Material

Web Material
