# Supplementary material for: Correlates of Peripheral Blood Mitochondrial DNA Content in a General Population
Source: Am J Epidemiol. 2015 Dec 24;183(2):138–46. doi: 10.1093/aje/kwv175 (PMC4706678; doi:10.1093/aje/kwv175)
Supplement: Web Material [file supp_183_2_138__index.html]

Correlates of Peripheral Blood Mitochondrial DNA Content in a General Population — Correlates of Peripheral Blood Mitochondrial DNA Content in a General Population — Web Material 

# Correlates of Peripheral Blood Mitochondrial DNA Content in a General Population

## Web Material

Web Material

- Web Material - Pdf file
