# Supplementary material for: Correlates of Peripheral Blood Mitochondrial DNA Content in a General Population
Source: Am J Epidemiol. 2015 Dec 24;183(2):138–46. doi: 10.1093/aje/kwv175 (PMC4706678; doi:10.1093/aje/kwv175)
Supplement: Web Material [file supp_kwv175_kwv175supp.pdf]

# Correlates of Peripheral Blood Mitochondrial DNA Content in a General Population

## Web Material

# WEB APPENDIX

## Methods

The diseases were coded according to the *International Classification of Diseases, Eighth Revison*. Inflammatory diseases included any infectious diseases (codes 001-139) or any inflammatory processes of any organ system, including nervous system (codes 320-324; 354), eye (codes 360-369), ear (codes 380-384), respiratory system (codes 460-466, 470-474, 480-486, 490, 513), cardiovascular system (codes 390-392, 420-422, 451), gastrointestinal system (codes 530, 535, 561, 563, 563.1), the oral cavity (codes 552.2, 522.5), urogenital system (codes 590-590.2, 601, 616), skin/subcutaneous tissue (codes 680-686) and osteomuscular system (codes 710-711, 720-721, 731-732, 734, 734.1). Diseases of blood and blood forming organs (codes 280-289) and neoplasms of lymphatic and haematopoietic tissue (codes 200-209) were considered as blood diseases. Neoplasms encompass codes from 140 to 239, which include malignant neoplasms (codes 140-209 (‘*cancer*’)).

Antiaggregation drugs included salicylates, adenosine diphosphate receptor inhibitors and/or adenosine reuptake inhibitors.

## Calculation of mtDNA content

The mtDNA content was calculated using qBase software (Biogazelle, Zwijnaarde, Belgium), as described in detail elsewhere (1). Briefly, each sample was amplified in a triplicate. First, we calculated mean threshold values (Ct) of individual triplicates and mean Ct values of each of the genetic sequence of all samples in all plates. The delta Ct was the difference between mean single triplet Ct value and mean Ct value averaged over all plates for each sequence. Relative quantities (RQ) with 100% primer efficiency equal $2^{\text{delta Ct}}$. Second, normalized relative quantities (NRQ) for each subject were calculated by dividing the corresponding RQ of each mitochondrial sequence with the RQ of the nuclear sequence. Finally, interrun calibration was performed on a gene to gene basis. Calibration factors are based on geometric means of NRQ of interrun calibrators. To calculate calibrated normalized relative quantities (CNRQ), sample NRQs on one plate were multiplied with the calibration factors. The final mtDNA content is the average CNRQ value of both mitochondrial sequences.

**Web Table 1.** Primer sequences and efficiencies for selected mitochondrial and nuclear amplification targets, FLEMENGHO, 2009–2013

| **Sequence/Gene** | **Location** | **Accession Number** | **Forward 5'-3'** | **Reverse 3'-5'** | **Efficiency (%)** |
|---|---|---|---|---|---|
| *MT-ND1* | M | NC_012920.1 | ATGGCCAACCTCCTACTCCT | CTACAACGTTGGGGCCTTT | 99.9 |
| *MTF3212/R3319* | M | NC_012920.1 | CACCCAAGAACAGGGTTTGT | TGGCCATGGGTATGTTGTTAA | 99.7 |
| *RPLP0* | N | NM_001002.3 | GGAATGTGGGCTTTGTGTTC | CCCAATTGTCCCCTTACCTT | 100 |

*MT-ND1,* mitochondrial encoded NADH dehydrogenase 1; *MTF3212/R3319* mitochondrial forward primer from nucleotide 3212 and reverse primer from nucleotide 3319; *RPLP0,* Ribosomal protein, large, P0; M, mitochondrial; N, nuclear. Accession numbers are from National Center for Biotechnology Information (www.ncbi.nlm.nih.gov).

**Web Figure 1.** Distribution of the relative mitochondrial DNA (mtDNA) content in the entire population, FLEMENGHO, 2009–2013.

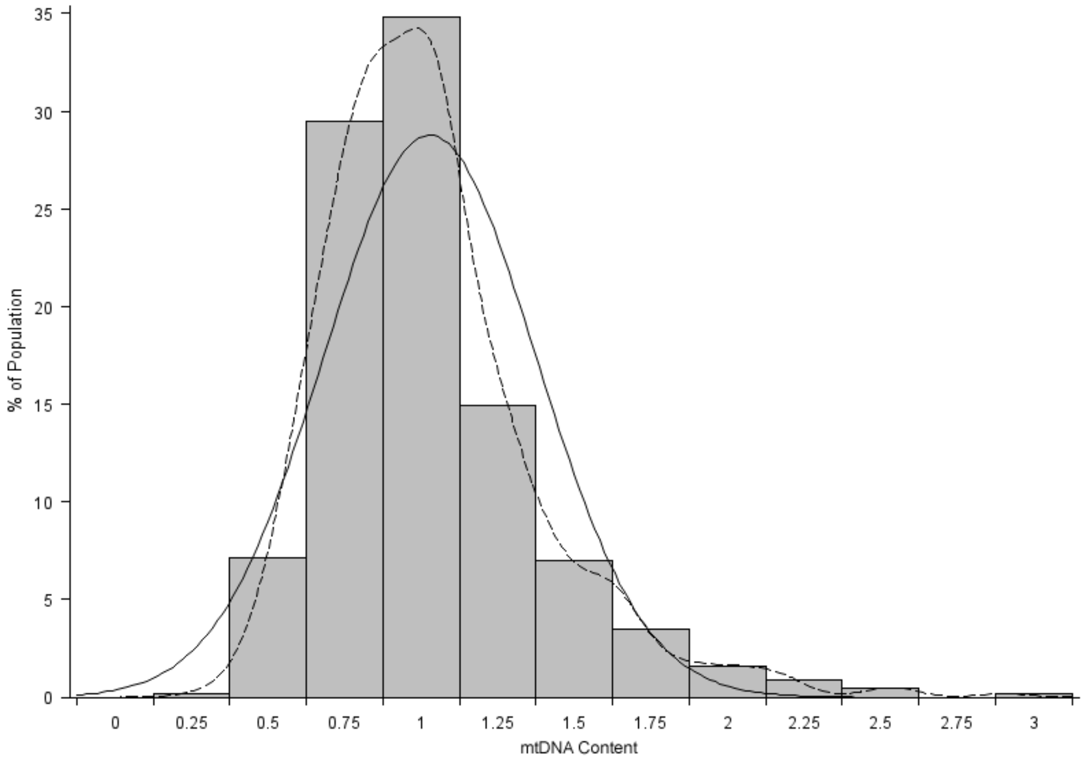


*Notes*: The curves represent the fitted normal (solid line) and Kernel (dashed line) density plots. Coefficients of skewness and kurtosis were 1.33 ($P < 0.01$) and 3.03, respectively.

**Web Figure 2.** Unadjusted mitochondrial DNA (mtDNA) content by age with the fitted quadratic curve in all subjects (panel A) and by sex (women, panel B; men, panel C), FLEMENGHO, 2009–2013.

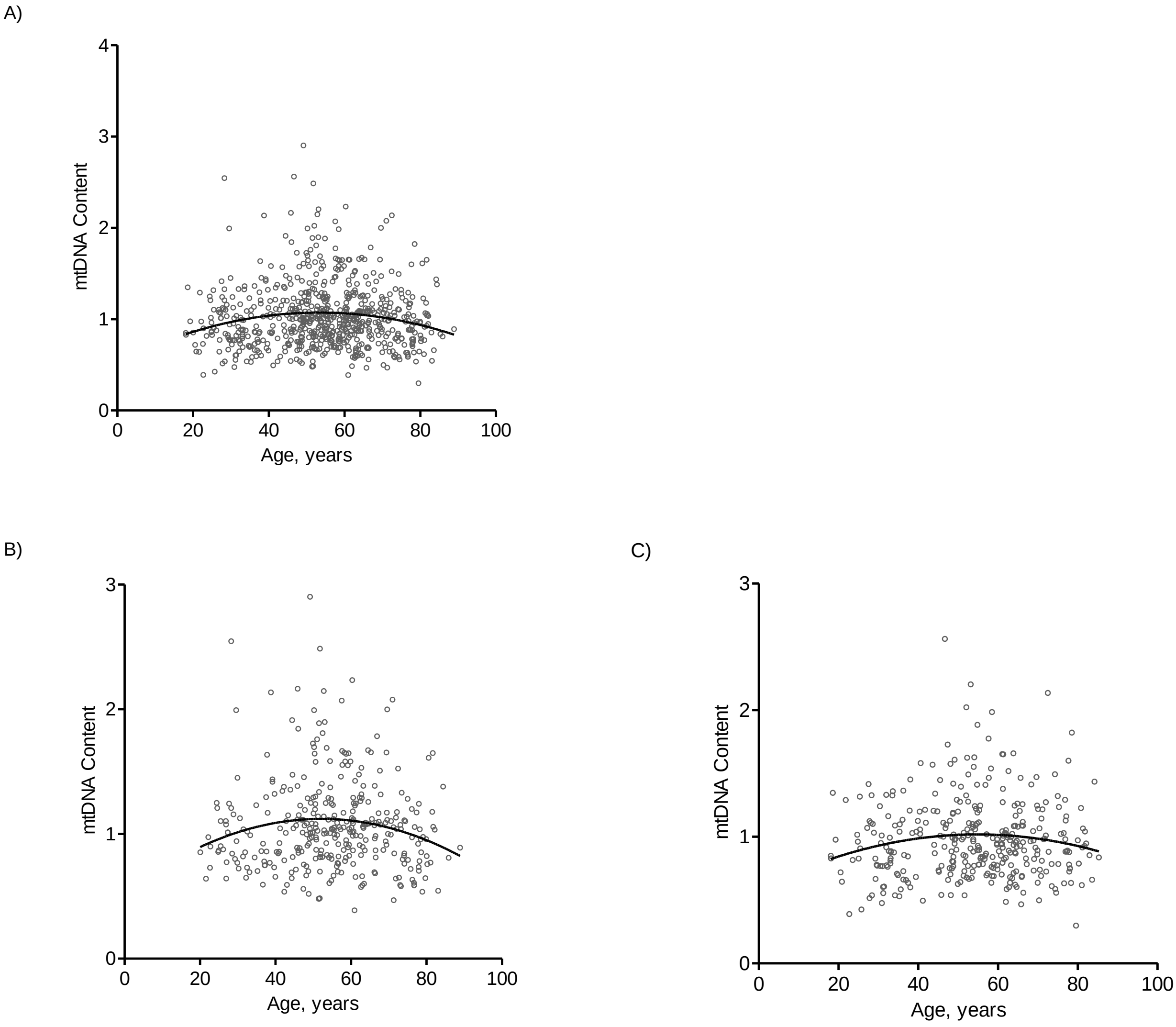

**Web Table 2.** Correlates[a] of mtDNA content in subjects without antiaggregation therapy ($n$ = 580), FLEMENGHO, 2009–2013

| Parameter | mtDNA Content | | | | |
|---|---|---|---|---|---|
| | Partial $r^2$ (%) | Parameter Estimate | Standard Error | 95% CI | *P* Value |
| Age, per year[b] | 2.24 | 0.017 | 0.006 | 0.005, 0.028 | 0.0023 |
| $Age^2$ | / | -0.00017 | 0.000054 | -0.0003, -0.0001 | 0.0018 |
| Female sex | 0.67 | 0.080 | 0.032 | 0.017, 0.14 | 0.012 |
| White blood cells (1.60 x $10^9$ cells/L)[c] | 4.46 | -0.094 | 0.014 | -0.12, -0.069 | <0.0001 |
| Platelets (56.8 x $10^9$ cells/L)[c] | 3.67 | 0.057 | 0.015 | 0.0283, 0.086 | 0.0001 |
| Intake of systemic hormone therapy | 0.47 | -0.092 | 0.053 | -0.20, 0.011 | 0.082 |
| Total adjusted $R^2$ (%) | 11.5 | | | | |

[a] The covariables considered for entry into the stepwise regression model were sex, age, body height, body weight, waist circumference, body mass index, systolic and diastolic blood pressure, plasma glucose, serum insulin, total cholesterol, serum creatinine, current smoking and drinking, systemic hormone therapy. We set the *P* values for covariates to enter and to stay in the regression models at 0.10 Variance inflation factors (VIF) were ≤1.30 for all explanatory variables. [b]Partial $r^2$ of age includes the partial $r^2$ of $age^2$. [c]Parameter estimates and corresponding standard errors and 95% CI for blood cell counts are expressed for a 1 SD increase in the explanatory variables. mtDNA, mitochondrial deoxyribonucleic acid; FLEMENGHO, The Flemish Study on Environment, Genes and Health Outcomes, CI, confidence interval; SD, standard deviation.

**Web Table 3.** Correlates[a] of mtDNA content in subjects without a previous history of blood diseases (*n* = 647), FLEMENGHO, 2009–2013

| Parameter | mtDNA Content | | | | |
|---|---|---|---|---|---|
| | Partial $r^2$ (%) | Parameter Estimate | Standard Error | 95% CI | *P* Value |
| Age, per year[b] | 2.16 | 0.017 | 0.005 | 0.007, 0.027 | 0.0008 |
| Age$^2$ | / | -0.0002 | 0.00005 | -0.0003, -0.0001 | 0.0004 |
| Female sex | 0.49 | 0.067 | 0.029 | 0.010, 0.12 | 0.022 |
| White blood cells (1.60 x $10^9$ cells/L)[c] | 4.26 | -0.092 | 0.014 | -0.12, -0.065 | <0.0001 |
| Platelets (56.8 x $10^9$ cells/L)[c] | 2.89 | 0.051 | 0.015 | 0.022, 0.080 | 0.0005 |
| Intake of systemic hormone therapy | 0.40 | -0.064 | 0.038 | -0.14, 0.010 | 0.091 |
| Total adjusted $R^2$ (%) | 10.2 | | | | |

[a] The covariables considered for entry into the stepwise regression model were sex, age, body height, body weight, waist circumference, body mass index, systolic and diastolic blood pressure, plasma glucose, serum insulin, total cholesterol, serum creatinine, current smoking and drinking, systemic hormone therapy. We set the *P* values for covariates to enter and to stay in the regression models at 0.10 Variance inflation factors (VIF) were ≤1.30 for all explanatory variables.[b]Partial $r^2$ of age includes the partial $r^2$ of age$^2$.
[c]Parameter estimates and corresponding standard errors and 95% CI for blood cell counts are expressed for a 1 SD increase in the explanatory variables. mtDNA, mitochondrial deoxyribonucleic acid; FLEMENGHO, The Flemish Study on Environment, Genes and Health Outcomes, CI, confidence interval; SD, standard deviation.

**Web Table 4.** Correlates[a] of mtDNA content in subjects without history of inflammatory diseases ($n$ = 659), FLEMENGHO, 2009–2013

| Parameter | mtDNA Content | | | | |
|---|---|---|---|---|---|
| | Partial $r^2$ (%) | Parameter Estimate | Standard Error | 95% CI | *P* Value |
| Age, per year[b] | 2.28 | 0.017 | 0.005 | 0.007, 0.027 | 0.0009 |
| Age$^2$ | / | -0.0002 | 0.00005 | -0.0003, -0.0001 | 0.0004 |
| Female sex | 0.57 | 0.074 | 0.029 | 0.017, 0.13 | 0.010 |
| White blood cells (1.60 x $10^9$ cells/L)[c] | 4.13 | -0.091 | 0.014 | -0.12, -0.064 | <0.0001 |
| Platelets (56.8 x $10^9$ cells/L)[c] | 2.73 | 0.049 | 0.014 | 0.022, 0.076 | 0.0006 |
| Intake of systemic hormone therapy | 0.59 | -0.11 | 0.051 | -0.21, 0.010 | 0.038 |
| Total adjusted $R^2$ (%) | 10.3 | | | | |

[a] The covariables considered for entry into the stepwise regression model were sex, age, body height, body weight, waist circumference, body mass index, systolic and diastolic blood pressure, plasma glucose, serum insulin, total cholesterol, serum creatinine, current smoking and drinking, systemic hormone therapy. We set the *P* values for covariates to enter and to stay in the regression models at 0.10 Variance inflation factors (VIF) were ≤1.30 for all explanatory variables.[b]Partial $r^2$ of age includes the partial $r^2$ of age$^2$.
[c]Parameter estimates and corresponding standard errors and 95% CI for blood cell counts are expressed for a 1 SD increase in the explanatory variables. mtDNA, mitochondrial deoxyribonucleic acid; FLEMENGHO, The Flemish Study on Environment, Genes and Health Outcomes, CI, confidence interval; SD, standard deviation.

**Web Table 5.** Correlates[a] of mtDNA content in subjects without history of cancer (*n* = 662), FLEMENGHO, 2009–2013

| Parameter | mtDNA Content | | | | |
|---|---|---|---|---|---|
| | Partial $r^2$ (%) | Parameter Estimate | Standard Error | 95% CI | *P* Value |
| Age, per year[b] | 2.13 | 0.017 | 0.005 | 0.007, 0.027 | 0.0009 |
| $Age^2$ | / | -0.0002 | 0.00005 | -0.0003, -0.00007 | 0.0004 |
| Female sex | 0.55 | 0.071 | 0.029 | 0.014, 0.12 | 0.014 |
| White blood cells (1.60 x $10^9$ cells/L)[c] | 3.98 | -0.092 | 0.014 | -0.12, -0.065 | <0.0001 |
| Platelets (56.8 x $10^9$ cells/L)[c] | 3.10 | 0.055 | 0.014 | 0.028, 0.082 | 0.0002 |
| Intake of systemic hormone therapy | 0.44 | -0.091 | 0.051 | -0.19, 0.009 | 0.075 |
| Total adjusted $R^2$ (%) | 10.2 | | | | |

[a] The covariables considered for entry into the stepwise regression model were sex, age, body height, body weight, waist circumference, body mass index, systolic and diastolic blood pressure, plasma glucose, serum insulin, total cholesterol, serum creatinine, current smoking and drinking, systemic hormone therapy. We set the *P* values for covariates to enter and to stay in the regression models at 0.10 Variance inflation factors (VIF) were ≤1.30 for all explanatory variables.[b]Partial $r^2$ of age includes the partial $r^2$ of $age^2$.
[c]Parameter estimates and corresponding standard errors and 95% CI for blood cell counts are expressed for a 1 SD increase in the explanatory variables. mtDNA, mitochondrial deoxyribonucleic acid; FLEMENGHO, The Flemish Study on Environment, Genes and Health Outcomes, CI, confidence interval; SD, standard deviation.

**Web Table 6.** Correlates[a] of mtDNA content in healthy participants ($n$ = 306), FLEMENGHO, 2009–2013

| Parameter | mtDNA Content | | | | |
|---|---|---|---|---|---|
| | **Partial $r^2$ (%)** | **Parameter Estimate** | **Standard Error** | **95% CI** | ***P* Value** |
| Age, per year | 2.13 | 0.004 | 0.001 | 0.001, 0.007 | 0.005 |
| Female sex | / | 0.030 | 0.041 | -0.050, 0.11 | 0.46 |
| White blood cells (1.60 x $10^9$ cells/L)[b] | 5.21 | -0.10 | 0.020 | -0.14, -0.061 | <0.0001 |
| Platelets (56.8 x $10^9$ cells/L)[b] | 2.94 | 0.068 | 0.020 | 0.029, 0.11 | 0.0018 |
| Intake of systemic hormone therapy | / | -0.058 | 0.056 | -0.17, 0.052 | 0.30 |
| Total adjusted $R^2$ (%) | 11.0 | | | | |

[a] The covariables considered for entry into the stepwise regression model were sex, age, body height, body weight, waist circumference, body mass index, systolic and diastolic blood pressure, plasma glucose, serum insulin, total cholesterol, serum creatinine, current smoking and drinking, systemic hormone therapy. We set the *P* values for covariates to enter and to stay in the regression models at 0.10 Variance inflation factors (VIF) were ≤1.30 for all explanatory variables.[b]Parameter estimates and corresponding standard errors and 95% CI for blood cell counts are expressed for a 1 SD increase in the explanatory variables. mtDNA, mitochondrial deoxyribonucleic acid; FLEMENGHO, The Flemish Study on Environment, Genes and Health Outcomes, CI, confidence interval; SD, standard deviation.

**Web Figure 3.** Mitochondrial DNA (mtDNA) content vs. neutrophil count (panel A; *r* = −0.23, *P* < 0.0001), monocyte count (panel B; *r* = −0.13, *P* = 0.0015), and lymphocyte count (panel C; *r* = −0.12, *P* = 0.0015) in multivariable−adjusted analyses, FLEMENGHO, 2009–2013.

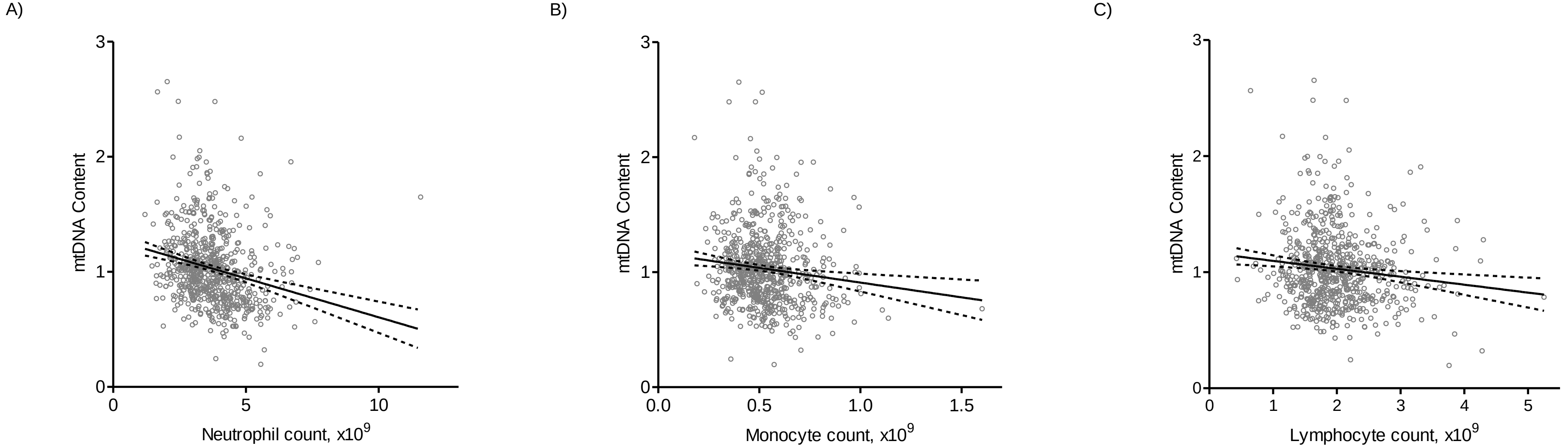


*Notes*: The solid and dashed lines represent the regression line and the 95% confidence interval, respectively. Analyses were adjusted for age, sex, platelet count, systemic hormone therapy, and family clusters.

**Web Table 7.** Multivariable-adjusted[a] correlations of mtDNA content with blood cells in subjects without a previous history of blood disease (*n* = 647), FLEMENGHO, 2009–2013

| Explanatory Variable | MtDNA Content | | | |
|---|---|---|---|---|
| | **Parameter Estimate** | **Standard Error** | **95% CI** | ***P* Value** |
| Platelets (56.8 x $10^9$ cells/L)[b] | 0.051 | 0.015 | 0.022, 0.080 | 0.0002 |
| White blood cells (1.60 x $10^9$ cells/L)[b] | -0.095 | 0.014 | -0.053, -0.032 | <0.0001 |
| Segmented neutrophils (1.17 x $10^9$ cells/L)[b] | -0.084 | 0.013 | -0.11, -0.059 | <0.0001 |
| Monocytes (0.17 x $10^9$ cells/L)[b] | -0.051 | 0.014 | -0.078, -0.024 | 0.0002 |
| Lymphocytes (0.66 x $10^9$ cells/L)[b] | -0.052 | 0.014 | -0.079, -0.025 | 0.0002 |
| Eosinophils (0.11 x $10^9$ cells/L)[b] | -0.002 | 0.013 | -0.027, 0.023 | 0.89 |
| Basophils (0.017 x $10^9$ cells/L)[b] | -0.014 | 0.014 | -0.041, 0.013 | 0.31 |

[a]Models were adjusted for sex, age, systemic hormone therapy, and family clusters. For white blood cells, adjusted model included platelet count. For platelets, adjusted model included white blood cell count. [b]Parameter estimates and corresponding SE and 95% CI are expressed for a 1 SD increase in the explanatory variables. FLEMENGHO, The Flemish Study on Environment, Genes and Health Outcomes; mtDNA, mitochondrial deoxyribonucleic acid; CI, confidence interval; SD, standard deviation.

**Web Figure 4.** Multivariable-adjusted mitochondrial DNA (mtDNA) content by indication for systemic hormone therapy (SHT) in women, FLEMENGHO, 2009–2013.

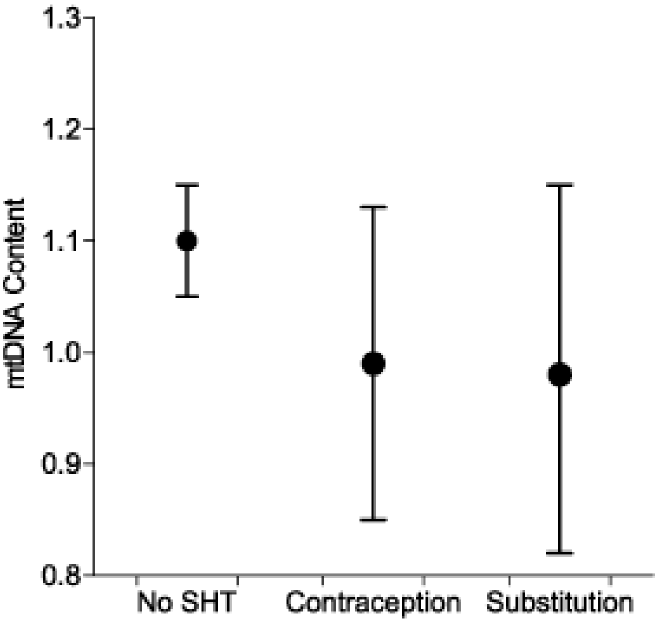


*Notes*: Models were adjusted for age, platelet count, white blood cell count and family clusters. The *P* value for comparison between women without SHT (*n* = 290) and women on SHT for contraception (*n* = 39) was 0.13. The *P* value for comparison between women without SHT and women on SHT for substitution (*n* = 18) was 0.16. Bars, 95% confidence interval.

## Web Reference

1. Hellemans J, Mortier G, De Paepe A, et al. qBase relative quantification framework and software for management and automated analysis of real-time quantitative PCR data. *Genome Biol.* 2007;8:R19.
